# Modeling competence development in the presence of selection bias

**DOI:** 10.3758/s13428-018-1021-z

**Published:** 2018-02-15

**Authors:** Sabine Zinn, Timo Gnambs

**Affiliations:** Leibniz Institute for Educational Trajectories, Wilhelmsplatz 3, 96047 Bamberg, Germany

**Keywords:** Competence development, Selection bias, Selectivity analysis, Longitudinal design

## Abstract

A major challenge for representative longitudinal studies is panel attrition, because some respondents refuse to continue participating across all measurement waves. Depending on the nature of this selection process, statistical inferences based on the observed sample can be biased. Therefore, statistical analyses need to consider a missing-data mechanism. Because each missing-data model hinges on frequently untestable assumptions, sensitivity analyses are indispensable to gauging the robustness of statistical inferences. This article highlights contemporary approaches for applied researchers to acknowledge missing data in longitudinal, multilevel modeling and shows how sensitivity analyses can guide their interpretation. Using a representative sample of *N* = 13,417 German students, the development of mathematical competence across three years was examined by contrasting seven missing-data models, including listwise deletion, full-information maximum likelihood estimation, inverse probability weighting, multiple imputation, selection models, and pattern mixture models. These analyses identified strong selection effects related to various individual and context factors. Comparative analyses revealed that inverse probability weighting performed rather poorly in growth curve modeling. Moreover, school-specific effects should be acknowledged in missing-data models for educational data. Finally, we demonstrated how sensitivity analyses can be used to gauge the robustness of the identified effects.

**Electronic supplementary material:**

The online version of this article (10.3758/s13428-018-1021-z) contains supplementary material, which is available to authorized users.

The conditions for successful learning and obstacles to academic achievement are among the most prevalent topics debated by educational specialists, psychologists, and policy makers alike. Therefore, several large-scale efforts, such as the Programme for International Student Assessment (PISA) or the Programme for the International Assessment of Adult Competences (PIAAC), have been initiated that allow the identification of individual and context factors fostering academic success. Although these cross-sectional studies provided important insights into, for example, the associations between motivations and academic achievement (Jansen, Scherer, & Schroeders, 2015), they are silent on within-person processes such as the development of cognitive abilities over time. These research questions require coherent measurements of competences across distinct educational stages (e.g., from elementary school to secondary school) and, thus, longitudinal data across an individual’s school career (Weinert et al., 2011). Only rather recently, large-scale assessments have been enriched by longitudinal components that allow for these types of analyses (e.g., Prenzel, Carstensen, Schöps, & Maurischat, 2006; Rammstedt, Martin, Zabal, Carstensen, & Schupp, 2017). By design, these studies are representative of their target population (e.g., 15-year-olds in grade 7 or above in PISA, or adults residing in a country aged 16 to 65 years in PIAAC) and, thus, strive for population-based inferences. In these studies, nonresponse poses a fundamental threat to the representativeness of the observed samples, especially in voluntary, low-stakes assessments, because certain respondents refuse to participate in a study as a whole or refuse to provide responses to selected items of the administered instrument. In longitudinal studies, this problem is even worse because repeated participation (i.e., at multiple measurement occasions) is expected from each respondent. However, in repeated measurement designs, it is not uncommon to observe pronounced dropout rates over the course of a study because of, for example, dwindling motivations, survey fatigue, or time constraints (see Helmschrott & Martin, 2014). This is particularly troubling if dropout does not occur randomly but is associated with distinct respondent characteristics. In these situations, statistical analyses that ignore the selection bias can lead to invalid inferences regarding effects on the population level under study. Although several articles have highlighted issues associated with missing values in longitudinal research (e.g., Enders, 2011; Muthén, Asparouhov, Hunter, & Leuchter, 2011), awareness of the problem has, as of yet, not diffused into mainstream psychological practice. Most longitudinal research still resorts to complete case analyses and implicitly assumes random dropout processes, without evaluating this assumption empirically. This article details and contrasts common statistical methods that can be used for modeling competence development in large-scale assessments when a nonrandom dropout mechanism is suspected and the propensity toward nonresponse over time presumably depends on the outcome variable under study. Particular emphasis is put on the clustered data structures that are typical in educational research, where, for example, students are nested within different schools. Readers are encouraged to replicate our work and to adapt it for their own research projects using the documented analysis syntax that is provided as a supplement to this article.

## Drivers of adolescent competence development: The data example

There is broad consensus that intelligence is a driving force behind higher mathematical competence, with correlations between the two constructs frequently exceeding .60 (e.g., Deary, Strand, Smith, & Fernandes, 2007; Kuncel, Hezlett, & Ones, 2004). However, motivational constructs such as intrinsic motivation or ability self-perceptions show incremental effects on domain-specific competences, even after controlling for general intelligence (Trautwein et al., 2012). Although numerous studies identified robust cross-sectional associations between competence, intelligence, and motivation, little is known about the relative contribution of cognitive and noncognitive factors to the growth of domain-specific competences over time. Only recently did a reanalysis of data from PISA 2003 show that among 15-year-old students, task-specific self-efficacy also predicted growth in mathematical competence within a year (Kriegbaum, Jansen, & Spinath, 2015). Because nonresponse was not addressed in this study, it is unknown to what degree a potential selection bias might have affected these results. We approach this issue by presenting a conceptual replication of the study and focus on the question of how to model competence development in the presence of selection bias. Our analyses make use of a representative sample of German students from the National Educational Panel Study (NEPS; Blossfeld, Roßbach, & von Maurice, 2011), who were assessed in grade 9 and again three years later. We examine the relative effects of general intelligence and mathematical self-concept on the growth of mathematical competences over three years.

In psychology, changes over time are typically analyzed using growth curve models (GCM; Curran, Obeidat, & Losardo, 2010; Liu, Rovine, & Molenaar, 2012). GCMs specify repeated measures of an outcome (i.e., mathematical competence in our example) as a function of time (and, potentially, various covariates), thus estimating growth trajectories. Because they comprise individual-specific random effects, individual differences in competences can be accounted for. Formally, a basic GCM can be written as

1 $$ {Y}_{ti}={\gamma}_0+{\gamma}_1\cdot {x}_{ti}+{u}_i+{e}_{ti} $$

where *Y*_*ti*_ represents the competence score for individual *i* at time *t* (with *t* = 1 . . . *T*), *x*_*ti*_ is the time indicator for individual *i* (in our case: 0 for the first and 1 for the second measurement point), γ_0_ and γ_1_ represent the growth parameters reflecting the intercept and the linear growth process across all individuals, *u*_*i*_ captures the random effect for individual *i* (i.e., the deviation from the average intercept γ_0_), and *e*_*ti*_ is a residual term. In our application, a common time effect is assumed for all individuals; therefore, no time-specific random effects are considered. The model in Eq. 1 can be comparably specified as a structural equation or a mixed effects model (Chou, Bentler, & Pentz, 1998; Curran et al., 2010; McCallum, Kim, Malarkey, & Kiecolt-Glaser, 1997). For sake of simplicity and without loss of generality, we will rely on mixed-effect models with manifest time variables in this article. A complication arises because in educational assessments such as the NEPS students are typically not randomly sampled. Rather, the sampling units are school classes or entire schools (see Steinhauer et al., 2015). Therefore, the residuals *e*_*ti*_ are unlikely to be independent for different students, and thus violate a basic assumption of GCM (Singer & Willett, 2003). To address this problem, the GCM in Eq. 1 can be extended by an additional random effect *v*_*s*_ that captures school-specific variations (see Eq. 2).

2 $$ {Y}_{tis}={\gamma}_0+{\gamma}_1\cdot {x}_{tis}+{u}_i+{v}_s+{e}_{tis} $$

The random effects *u*_*i*_ and *v*_*s*_, as well as the model residual *e*_*tis*_, are assumed to follow a normal distribution with zero mean. Moreover, the latter variable is uncorrelated to the former ones. The model in Eq. 2 can easily be extended to include additional covariates, predicting the change in competence over time as well as interactions with time that represent moderating influences on the studied change trajectory. In our analyses, we focused on the effects of reasoning (*REAS*) as an indicator of general intelligence and mathematical self-concept (*SC*) on competence development in mathematics (*MATH*) over time (*TIME*). Thus, the formal representation in Eq. 3 gives the analysis model pertaining to our research question.

3 $$ {MATH}_{tis}={\gamma}_0+{\gamma}_1\cdot {TIME}_{tis}+{\gamma}_2\cdot {REAS}_{is}+{\gamma}_3\cdot {SC}_{is}+{\gamma}_4\cdot {TIME}_{tis}\cdot {REAS}_{is}+{\gamma}_5\cdot {TIME}_{tis}\cdot {SC}_{is}+{u}_i+{v}_s+{e}_{tis} $$

This model is estimated using data of the NEPS (Blossfeld et al., 2011) that suffers from panel attrition and wave nonresponse (Steinhauer & Zinn, 2016). Particularly, competence measurements exhibit increasing missing rates in progressive assessment waves.

## Missing-data mechanisms

Generally, three kinds of nonresponse mechanisms are distinguished (Goldberger, 1981; Rubin, 1976): *missing completely at random* (MCAR), or random selection; *missing at random* (MAR), or nonrandom explicit selection; and *missing not at random* (MNAR), or nonrandom incidental selection. In the first case (MCAR), nonresponse occurs randomly and is not associated with (observed or unobserved) respondent characteristics or context factors. In the second case (MAR), the propensity toward nonresponse can be fully explained by the variables observed from the studied population. An example is the smaller propensity of working people to participate in a telephone interview conducted during the workday. This is in contrast to MNAR, where the probability of a missing value in a variable depends either on the measured variable itself or on some other unobserved factor correlated with it. For instance, high earners are less likely to divulge their income and to participate in financial surveys. In our data example, MAR processes might result for the second measurement of mathematical competence, because low-ability students with inferior test performance at the first assessment are likely to have a higher propensity to drop out of the study. At the same time, various unobserved variables, such as interest in the study domain or having enough spare time (cf. Helmschrott & Martin, 2014), can be driving forces for survey participation and, thus, contribute to MNAR processes. It is important to note that the distinction between MAR and MNAR is not exclusively a characteristic of the data, but also depends on the statistical analyses (see Graham, 2009). Assume that the initial competence levels are the cause for the missingness of mathematical competence at the second wave (as described above). If the observed competence scores from Wave 1 (or a time effect, as in Eq. 3) were included in the statistical analyses, then mathematical competence in Wave 2 would be conditioned on the previous competence estimates; thus, the model would be MAR. However, if the competence scores from Wave 1 were excluded from the analyses, then the missingness problem would become MNAR, because the cause of missingness would not be included in the statistical model.

**Fig. 1:**
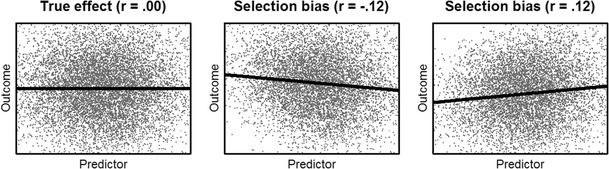
Example of selection bias in a simulated sample with regression lines, for a response rate of 80%. The correlations between the outcome and the response group were .50 (middle panel) and – .50 (right panel)

If a selection pattern is caused by an MCAR process, the dropout process can be ignored in the statistical modeling approach without producing biased results; thus, complete case analyses do not bias statistical tests. In contrast, MAR and MNAR processes must be specifically addressed in the statistical analyses to avoid invalid inferences. Ignoring the nonresponse mechanism (as is the case with listwise deletion) would produce misleading (if not erroneous) results. For example, if students with lower interest in the tested domain (i.e., mathematics) have weaker growth rates and, at the same time, exhibit a higher propensity to drop out of the study, ignoring this pattern would yield an overestimation of competence growth. Similar, if students with a high workload due to demanding vocational training are less likely to participate but, at the same time, show stronger growth rates, a model not accounting for this process would underestimate competence growth over time. To emphasize the consequences of ignoring the dropout mechanism, consider the artificial example in Fig. 1: For a simulated sample of 10,000 participants, the true correlation between an outcome *Y* and a predictor *X* is 0 (left panel). Assume that nonrandom dropout leads to a nonresponse rate of 20% (in empirical studies, these sometimes even approach 50%; Chatfield, Brayne, & Matthews, 2005) and that both variables are correlated at .50 with the response probability. Then, the observed sample would exhibit a correlation between *Y* and *X* of *r* = – .12. In the case of a negative correlation between the outcome and the response probability, the observed correlation in the sample would be *r* = .12. Thus, depending on the selection mechanism, either a positive or a negative correlation would arise, despite the two variables being uncorrelated in the full sample. This example demonstrates the adverse effects of selection bias on observed sample statistics. For MAR, several established methods exist that have been shown to produce unbiased parameter estimates and standard errors of population effects. Examples are multiple imputation (Rubin, 1987; Van Buuren, 2011, 2012), likelihood-based approaches such as full-information maximum likelihood (Allison, 2001, 2003; Enders, 2010; Enders & Bandalos, 2001), and inverse probability weighting (Kalton & Flores-Cervantes, 2003; Valliant, Dever, & Kreuter, 2013). However, the situation is more complicated if the response probability depends on the outcome *Y* itself or on some unobserved variables. In both cases, the selection process is nonrandom (MNAR).

## Missing-data models for MNAR

Generally, two classes of methods exist that can be used under MNAR (Molenberghs & Kenward, 2007; Rubin, 1987). The first class uses external information for the variables with missing values, such as findings from previous studies, expert knowledge, or information recorded by national agencies (e.g., official statistics), to compensate for distortions in the model likelihood. The second class explicitly models the missing-data mechanism by assuming a specific missing-data model. The first class is clearly the one that should be preferred, because it uses the proper distribution for the missing values. However, often reliable external information to compensate for missing values might not be accessible or available, or it simply might not exist. Therefore, the second approach poses the only viable solution in many applied contexts. Two common likelihood-based approaches for modeling MNAR are selection and pattern mixture models (Enders, 2011; Little, 1995, 2008; Muthèn et al., 2011):

Let *Y* = (*y*_*ij*_)_*i* = 1, …, *NT*; *j* = 1, …, *p*_ be the *NT* × *p* data matrix for *N* individuals, *p* variables, and *T* time points, and let *R* = (*r*_*ij*_)_*i* = 1, …, *NT*; *j* = 1, …, *p*_ be the *NT* × *p* indicator matrix that specifies whether a value of *Y* is observed (i.e., *r*_*ij*_ = 0 if *y*_*ij*_ is missing, and *r*_*ij*_ = 1 otherwise). In our data example, there are two measurement waves, with incomplete data for the second wave. Then, the full data likelihood can be written as *f*(*Y*, *R* | θ, ψ), where θ contains the parameters of the analysis model and ψ the parameters of the missing-data model. The selection model and the pattern mixture model differ in the factorization of the full data likelihood, and thus in their handling of missing values. The selection model factorizes the full data likelihood into Eq. 4, whereas the pattern mixture model uses Eq. 5. Here, the stacked vector *Y*_obs_ contains the observed values of *Y*, and the stacked vector *Y*_mis_ contains the missing values of *Y*. Pattern mixture models can also be formulated as latent class models that specify different latent classes for each dropout pattern (Gottfredson, Bauer, & Baldwin, 2014), as given in Eq. 6 for *K* latent classes.

4 $$ f\left(Y,R|\uptheta, \uppsi \right)=f\left(Y|\uptheta \right)\cdot f\left(R|Y,\uppsi \right) $$

5 $$ f\left(Y,R|\uptheta, \uppsi \right)=f\left(Y|R,\uptheta \right)\cdot f\left(R|\uppsi \right)=f\left({Y}_{obs}|\uptheta \right)\cdot P\left(R=1|\uppsi \right)+f\left({Y}_{mis}|\uptheta \right)\cdot P\left(R=0|\uppsi \right) $$

6 $$ f\left({Y}_{it},{R}_{it}|\uptheta, \uppsi \right)=\sum \limits_{k=1}^KP\left({c}_{it}=k\right)\cdot f\left({Y}_{it}|{c}_{it}=k,\uptheta \right)\cdot f\left({R}_{it}|{c}_{it}=k,\uppsi \right) $$

Thus, the selection model specifies the marginal distribution of *Y* conditioned on the missing-data mechanism. In contrast, the pattern mixture model specifies the full data likelihood as a mixture over response patterns or unobserved factors driving the missing-data mechanism. A limitation of both approaches, the selection model *and* the pattern mixture model, is that they make untestable distributional assumptions. In the selection model the distribution *f*(*R* | *Y*, ψ) has to be specified, whereas in the pattern mixture model the distribution *f*(*Y*_*mis*_ | θ) needs to be stated. For example, for *f*(*R* | *Y*, ψ) a probit model might be assumed, and for *f*(*Y*_*mis*_ | θ) a normal distribution can be specified. The fact that both approaches rely on untestable assumptions makes it advisable to estimate both types of models and to compare results from different modeling strategies.

## The need for sensitivity analyses

Over the last decade, an increasing awareness has emerged among psychometricians and applied statisticians in general (especially among biostatisticians) that sensitivity analyses of incomplete-data models are an essential element in longitudinal data analyses (e.g., Carpenter & Kenward, 2012; Enders, 2011; Little et al., 2012; Molenberghs, Verbeke, & Kenward, 2008; Muthén et al., 2011). Sensitivity analyses evaluate the impact of nonresponse and different missing-data models on observed sample effects. Because missing-data handling methods are based on different assumptions (of which many are empirically untestable), it is mandatory to evaluate the degree to which the hypothesized missing-data process and the respective missing-data modeling strategy affect the estimated model parameters. For example, if data with MNAR are incorrectly treated as MAR or MCAR, the misspecified missing-data process is likely to produce biased parameters and standard errors (e.g., Li, Chen, Cui, & Liu, 2017). It should be noted that it is impossible to decide on an empirical basis whether a missing-data process is MAR or MNAR, because for every MNAR model there is a MAR model with an identical fit to a set of observed outcomes (see Molenberghs, Beunckens, Sotto, & Kenward, 2008). Moreover, since different ways of modeling MAR or MNAR incorporate different assumptions (e.g., regarding unobserved distributions), applied researchers are well-advised to conduct sensitivity analyses to evaluate the robustness of their derived parameters with regard to missing-data handling choices.

**Fig. 2:**
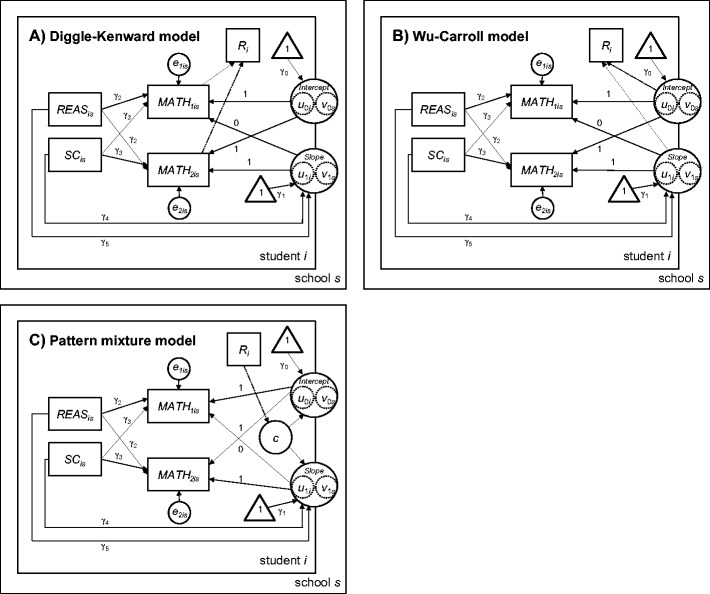
Path diagram of the selection and pattern mixture models. The dashed lines mark the selection equation for modeling participation at the second measurement point, indicated by the dummy-coded indicator R_i_ (A and B), or for determining the membership within one of the two latent dropout classes *c* (C)

In the present application, we adopted two popular selection models: those introduced by Diggle and Kenward (1994) and Wu and Carroll (1988). The *Wu–Carroll model* (WC) specifies a probit link to relate the dropout probability *f*(*R*| *Y*, ψ) to the analysis model, whereas the *Diggle–Kenward model* (DK) uses a logit specification. Moreover, in the DK the missing probability at time *t* depends on the past and the current individual growth trajectory, whereas in the WC this probability depends on an individual’s overall growth trajectory (see Fig. 2). Thus, at a given time point, *f*(*R*| *Y*, ψ) also acknowledges future observations. Both selection models allow for individual- and group-specific random effects, such as school effects, in the analysis model (see Eq. 2), but they consider only individual-specific effects in the selection equation. As an example of a *pattern mixture model*, we used Little’s (1995) latent-class model, which specifies different dropout groups over which the outcome distribution is a (latent) mixture, with *f*(*Y*_*mis*_ | θ) taken to be normal. Latent-class membership is defined by a multinomial logistic regression using dropout dummy variables as covariates. The model does not specify individual- or group-specific random effects when assigning class membership, but both types of random effects are possible in the analysis model. A formal description of these models as they pertain to our data example is given in the supplement. Under the MAR assumption, we used the *full-information maximum likelihood* (FIML) approach, a *multivariate-imputation-by-chained-equations method* (MI) that imputes competence data from a linear model (Van Buuren & Groothuis-Oudshoorn, 2011), and *inverse probability weighting* (Wooldridge, 2007). Each of these approaches makes it possible to consider the cluster structure of the data by allowing for school-specific random effects.

## Present study

We applied sensitivity analyses to the responses of a representative sample of German students, to evaluate how different missing-data models affect the estimated effects of self-concept and general intelligence on the growth of mathematical competence over three years. Thus, the results for the same analysis model for competence development are compared under different MAR and MNAR data schemes. The properties of the MAR and MNAR methods studied in this article, together with related statistical software (without claiming to be exhaustive), are summarized in Table 1.

**Table 1:** Sensitivity analyses for attrition in longitudinal, multilevel settings

Missing-Data Model	Individual- and Group-Specific Random Effects	Selection Variables Possible	Dependency on Time Trajectory	Statistical Software (Selected)
*Missing at random*				
Full-information maximum likelihood (FIML)	Both^a^	yes, for computing the model’s correlation matrix^b^	overall	Mplus, R (sem, lavaan, openMx), Stata (sem)
Multivariate imputation by chained equations (MI)	Either individual- or group-specific random effect	yes, in imputation model	overall	Mplus^c^, R (mice), Stata (ice)
Inverse probability weighting (WE)	Both	yes, in the response model yielding the weights	overall	Mplus, Stata (mixed, gllamm)^d^
*Missing not at random*				
Diggle–Kenward selection model (DK)	No individual- or group-specific effects in selection equation, but both in the analysis model	yes, in the selection model	past and present	Mplus Stata (gllamm)^e^
Wu–Carroll selection model (WC)	Individual-specific effect in selection equation, both in the analysis model	yes, in the selection model	overall	Mplus Stata (gllamm)^e^
Pattern mixture model (PM)	No individual- or group-specific effects for assigning latent groups, but both in the analysis model	no	overall	Mplus^e,f^

^a^Mplus facilitates the modelling of individual and group-specific effects, whereas the related R and Stata functions only allow the modelling of individual-specific effects. ^b^Only Mplus implements this feature, but solely for 1-level models. ^c^Mplus offers multiple imputation analysis options as well; however, the specification of the imputation model is hidden from the user. Thus, it is not possible to implement the chained regression approach as in mice or ice. ^d^So far, R does not explicitly allow inverse probability weights in random effects models. ^e^R can also be used to implement this kind of missing data models, however, this requires to write own estimation routines from scratch since (up to now) these models are not part of R’s officially contributed packages. ^f^Stata does not offer a single command for estimating this model, but the related routines may be implemented using Stata and its programming language Mata

## Method

### Participants

We used data from the longitudinal National Educational Panel Study (NEPS), which follows German students across their school careers (see Blossfeld et al., 2011). In 2010, a representative sample of students in lower secondary education from grade 9 was drawn across the country (see Steinhauer et al., 2015, for details on the sampling procedure). Students were tested in small groups at their respective schools by a professional survey institute. In sum, competence scores of 13,417 students (6,664 girls) in 538 schools were available. Their mean age was *M* = 14.92 (*SD* = 0.62) years. Three years later (i.e., in 2013), all students who did not refuse further participation were invited to a follow-up assessment. The students who remained at their original school were retested in the school context (3,651 students), whereas students who had switched school or left school altogether were tracked and individually tested at home (1,641 students). Thus, 8,125 of the students measured at Time 1 did not participate at Time 2 (i.e., almost 61% of the original sample). Further information on the data collection process, including the survey execution and the tracking of respondents, is provided in Steinhauer and Zinn (2016) and on the project website (www.neps-data.de).

### Instruments

*Mathematical competence* was measured at both measurement occasions with achievement tests that were specifically constructed for administration in the NEPS (see Neumann et al., 2013). To measure the competences with greater accuracy, different tests including either 22 or 29 items (with multiple choice or short constructed response formats) that were targeted at the competence level of the average student in the respective age group were administered in the two waves. All tests were scaled using models of item response theory (see Pohl & Carstensen, 2013). Competence scores were estimated as weighted maximum likelihood estimates (Warm, 1989) and linked across time to allow for valid mean level comparisons across the two measurement waves (see Fischer, Rohm, Gnambs, & Carstensen, 2016). The two tests exhibited IRT-based (marginal) reliabilities (Adams, 2005) of .79 and .75. Further details regarding the psychometric properties of these tests (including measurement invariance across measurement occasions and assessment contexts) are summarized in Duchhardt and Gerdes (2013) and Fischer, Rohm, and Gnambs (2017).

*Mathematical self-concept* was measured in grade 9 with three items (e.g., “I have always been good in math”) on four-point response scales from 1 = *does not apply at all* to 4 = *applies completely*. The composite reliability (McNeish, 2017) was good with ω_total_ = .90.

*Reasoning* was measured in grade 9 with a matrices test including 12 items (see Brunner, Lang, & Lüdtke, 2014). Because the figural content of these matrices is prototypical for fluid intelligence, matrices tests are good proxies for general intelligence (Wilhelm, 2005). Each item consisted of several fields with geometrical elements that followed a logical rule. Participants had to identify the underlying rule and select the correct element from a series of available response options. The number of correctly solved items served as an indicator of students’ reasoning abilities. On average, the participants correctly solved *M* = 8.65 (*SD* = 2.46) items. The categorical omega total reliability (Green & Yang, 2009) of this measure was ω_total_ = .74.

### Modeling strategy

The growth curve model in Eq. 3 was estimated under different missing-data schemes. In these analyses, the time effect (*TIME*) was coded as 0 and 1 for the first and second measurement occasion, respectively. The two predictors of growth, mathematical self-concept (*SC*) and reasoning (*REAS*), were *z*-standardized across all students. Before estimating our substantive model in Eq. 3, selectivity analyses examined potential predictors of nonresponse at the second measurement occasion. Selection variables identified in these analyses were subsequently used to model nonresponse under different missing-data schemes.

#### MCAR model

We estimated one MCAR model using listwise deletion (LWD). However, in our case a MCAR assumption justifying a complete case analysis is rather unlikely. Indeed, Little’s (1988) test rejected the MCAR hypothesis. However, LWD was used as a benchmark to highlight deviations originating from the MCAR assumption.

#### MAR models

We considered three different MAR models. First, the model in Eq. 3 was estimated using full maximum likelihood (FIML) without acknowledging the selection mechanism. Although it is theoretically possible to consider selection variables in FIML (see Graham, 2003), to our knowledge current software implementations are limited to single level models. Hence, in our analyses FIML does not accommodate information about the selection process. Second, we used a multivariate imputation by chained equations method (Van Buuren & Groothuis-Oudshoorn, 2011) that accounts for within- and between-individual variation in the imputation model (MI). However, it neglects variation between schools. The reason is that prevalent software (see Table 1) only implements two-level imputation methods (e.g., Grund, Lüdtke, & Robitzsch, 2016). In this situation, it is best to focus on the level explaining more variance as indicated by the intra-class correlation. In our case, this is the individual level with 63%. On the school level, we only measure an intra-class correlation of 31%. Third, inverse probability weights (WE) were calculated using the identified selection variables (Wooldridge, 2007). Theoretically, WE would allow for considering any kind of cluster structure when computing response probabilities and, hence, inverse probability weights. However, the data problem at hand prevented considering individual random effects, since only one observation per individual indicated participation. Furthermore, even though theoretically the school level may be included when predicting response probabilities, our data allowed for predicting random intercepts for only 85% of the schools, because of identification issues. Thus, we derived inverse probability weights without acknowledging the multilevel data structure.

#### MNAR models

We considered three MNAR models that have previously been described in more detail: the Diggle–Kenward selection model (DK), the Wu–Carroll selection model (WC), and the pattern mixture model of Little (PM). Note that for PM in the group of nonrespondents no change in mathematical competence can be estimated because no observations were available for this group at the second measurement point. None of the three models implemented a school-specific random effect in the selection equation or for the assignment of individuals to latent classes (cf. Fig. 2). Only WC specifies an individual effect in the selection model, whereas DK and PM condition participation and group attendance on the preceding outcome values. A formal description of the mathematical models is given in the supplement.

#### Model comparisons

The identification of the model that describes the data best among all of the models considered would require a unique model selection criterion. Common criteria such as the Bayesian information criterion (BIC; Schwarz, 1978) or the Akaike information criterion (AIC; Akaike, 1974) could not be used, since the dimensionality of the dependent variable varied between models. For example, the dimension of the dependent variable in an MI model equals the number of observed and unobserved cases, but in a weighted model the dimension of *Y* corresponds to the number of observed values. In our view, cross-validation and out-of-sample-selection seem to be promising options. However, to the knowledge of the authors, right now no appropriate methodology exists that could be applied coherently to all missing-data models. Because the design and implementation of such a methodology is beyond the scope of this article, we limited our model comparisons to the estimated model parameters of the analysis model.

### Statistical software and analyses syntax

Multiple imputations were conducted with mice version 2.30 (Van Buuren & Groothuis-Oudshoorn, 2011). The LWD and MI models were estimated with lmer version 1.1–13 (Bates, Maechler, Bolker, & Walker, 2015). Sampling weights were derived and the weighted growth curve model was estimated by Stata version SE 13.0. Finally, we used Mplus version 8 (Muthén & Muthén, 1998–2017) to estimate the FIML, DC, WC, and PM models. The code for our analyses is available at https://github.com/bieneSchwarze/PaperModelingSelectionBias.

## Results

**Fig. 3:**
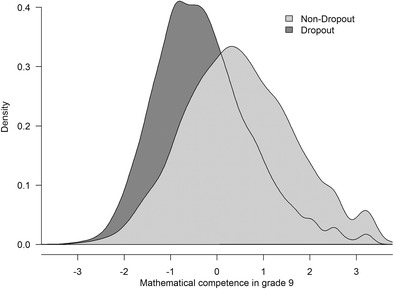
Distribution of mathematical competence in grade 9 by dropout group

The pairwise bivariate correlations between all measures are summarized in Table 2. As expected, mathematical competence showed moderate stability over three years (*r* = .70, *p <* .001). Moreover, mathematical self-concept and reasoning exhibited longitudinal associations with the second measurement of mathematical competence (both *r* = .41, *p* < .001). However, a fundamental problem with these (and similar) analyses is the pronounced nonresponse at the second measurement point, since about 61% of the initial sample did not participate in the second wave. More seriously, those who dropped out had significantly lower competencies in grade 9 than did the participants with responses at both measurement occasions (see Fig. 3). Thus, data were missing not completely at random.

**Table 2:** Means, standard deviations, and bivariate correlations between study variables

		*M*	*SD*	1.	2.	3.	4.	5.	6.	7.	8.	9.	10.
1.	Mathematical competence at *t = 1* (*MATH*_*1*_)	0.037	1.281										
2.	Mathematical competence at *t = 2* (*MATH*_*2*_)	1.081	1.111	.702									
3.	Self-concept (*SC*)	2.522	0.921	.347	.405								
4.	Reasoning (*REAS*)	8.654	2.457	.494	.408	.222							
5.	Sex ^a^	0.497	0.500	– .159	– .245	– .263	– .024						
6.	Migration ^b^	0.256	0.436	– .188	– .160	– .037	– .144	.015					
7.	Age (in years)	14.92	0.625	– .255	– .221	– .053	– .213	– .094	.154				
8.	Assessment mode ^c^	0.687	0.464	– .490	– .361	– .087	– .328	– .071	.097	.273			
9.	Basic secondary school ^d^	0.238	0.426	– .368	– .253	.005	– .355	– .069	.174	.256	.377		
10.	Intermediate secondary school ^d^	0.213	0.409	– .070	– .170	– .017	.033	– .004	– .029	.011	.351	– .290	
11.	Remaining school types ^d^	0.200	0.400	– .192	– .169	– .032	– .093	– .005	– .012	.031	.132	– .279	– .260

All correlations are significant at *p* < .001. On the basis of pairwise complete observations. Basic secondary school = “Hauptschule,” Intermediate secondary school = “Realschule.” ^a^Coded as 0 = boys and 1 = girls; ^b^Coded as 0 = no migration background and 1 = with migration background; ^c^Coded as 1 = tested in schools and 0 = tested individually at home; ^d^Dummy coded with upper secondary school (= “Gymnasium”) as reference category

### Selectivity analyses

We suspected that students did not drop out randomly over time. Therefore, predictors of nonresponse at the second measurement occasion were identified by regressing dropout (coded 0 = no dropout, 1 = dropout) on the variables in our analysis model (see Eq. 3) as well as on various individual and context variables that were assumed to influence the propensity to participate (see Table 3). First and foremost, school-leavers who were about to be tested individually at home were significantly more likely to refuse further participation than were students who remained in their original schools (*B* = 5.04, *p* < .001). Moreover, proportionally more dropout was observed for students with lower mathematical competence (*B* = – 0.14, *p* < .005) and self-concept (*B* = – 0.08, *p* < .05) in grade 9. Finally, girls and students attending upper secondary schools had a significantly higher reparticipation propensity than did boys or students visiting other school types. In contrast, students’ age or migration background showed no relevant effects. Consequently, at least partially, the nonresponse at the second competence measurement was driven by a MAR process. Therefore, the variables driving the selection process were included in various missing-data models (i.e., WE, MI, DK, and WC) for the estimation of the growth curve in Eq. 3.

**Table 3:** Logit regression analysis for nonresponse at the second measurement point

	*B*	95% CI
Intercept	– 1.941^*^	[– 2.451, – 1.431]
Reasoning	– 0.016	[– 0.096, 0.064]
Self-concept	– 0.079^*^	[– 0.151, – 0.006]
Sex^a^	– 0.185^*^	[– 0.322, – 0.049]
Migration^b^	0.039	[– 0.120, 1.992]
Age	– 0.159	[– 0.291, – 0.027]
Assessment mode^c^	5.037^*^	[ 4.738, 5.336]
Basic secondary school^d^	0.750^*^	[– 0.043, 1.544]
Intermediate secondary school^d^	1.290^*^	[ 0.314, 2.217]
Remaining school types^d^	0.606^*^	[– 0.207, 1.418]
Competence score	– 0.137^*^	[– 0.223, – 0.052]
Competence score squared	– 0.002	[– 0.031, 0.027]
Random effect (*SD*)	2.890	[ 2.625, 3.182]

*N* = 13,417. Dependent variable is dropout (coded as 1 = dropout and 0 = no dropout). ^a^Coded as 0 = boys and 1 = girls. ^b^Coded as 0 = no migration background and 1 = with migration background. ^c^Coded as 0 = tested in school and 1 = tested individually at home. ^d^Dummy-coded with upper secondary school (= “Gymnasium”) as reference category. Reasoning and self-concept were *z*-standardized. ^*^p < .05

### Growth curve modeling under different missing-data mechanisms

**Fig. 4:**
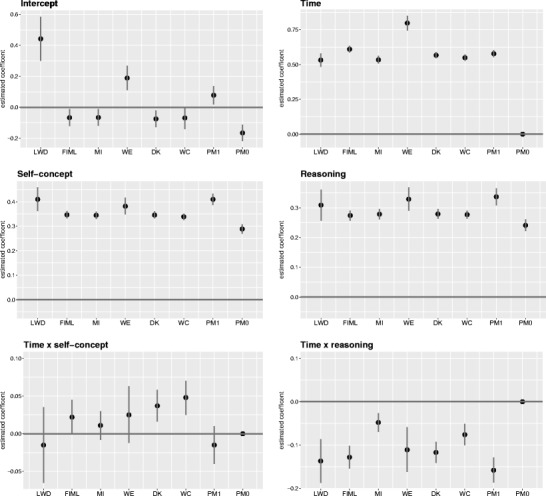
Estimated coefficients with 95% confidence intervals for the analysis model. LWD = listwise deletion; FIML = full-information maximum likelihood; MI = multivariate imputation via chained equations; WE = inverse probability weighting; DK = Diggle–Kenward selection model; WC = Wu–Carroll selection model; PM1/PM0 = pattern mixture model with two latent classes for all-time participants/dropout cases

**Fig. 5:**
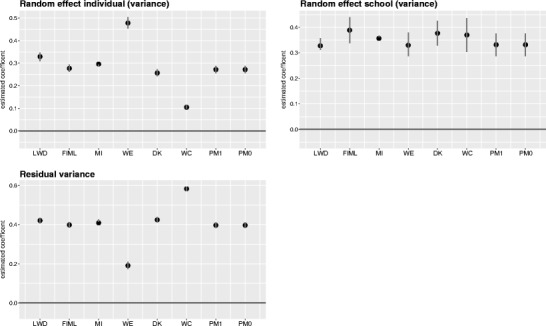
Estimated variance components, with 95% confidence intervals. LWD = listwise deletion; FIML = full-information maximum likelihood; MI = multivariate imputation via chained equations; WE = inverse probability weighting; DK = Diggle–Kenward selection model; WC = Wu–Carroll selection model; PM1/PM0 = pattern mixture model with two latent classes for all-time participants/dropout cases (for identification purposes, the variances are assumed to be equal for PM1 and PM0)

To analyze the effect of reasoning and self-concept on the growth of mathematical competence across three years, we estimated the growth curve model in Eq. 3 under different missing-data schemes. Coefficient plots for all models, with 95% confidence intervals, are given in Figs. 4 and 5 (the respective parameter estimates are available in the supplement).

#### Growth parameters

The growth of mathematical competence over three years is represented by the mean intercept and the linear time effect (γ_0_ and γ_1_ in Eq. 3), given in the upper panels of Fig. 4. At a first glance, the overall pattern of results does not indicate any single conclusion. However, differences between the estimates can be explained by distinct model assumptions. The intercept reflects students’ mean mathematical competence in grade 9 (after controlling for the effects of self-concept and reasoning). Particularly noticeable are the strongly deviating estimates under LWD and WE. We have previously shown that there is increased dropout among students with lower competence scores in grade 9. Ignoring this aspect in LWD leads to an overestimation of the overall mean score. WE faces a similar problem, because the same weight is applied to each individual observation, irrespective of the time point. However, weights compensate for the surplus of dropouts among underperforming students in grade 9 such that these students are assigned larger weights. This also results in an overestimated intercept. The higher mean intercept for nondropouts in the PM (model PM1) results from the fact that the students who participated at both waves had, on average, higher competence scores than did dropouts (model PM0). In contrast, FIML, MI, and the two selection models converge on similar estimates of the intercept.

In our specification, the time effect marks the average linear change across three years. All models estimated significant competence gains in mathematics over the three years, ranging from .53 to .78 (see Fig. 4). The estimates of the MAR models FIML and MI, as well as the estimates of the MNAR models DK, WC, and PM, were rather similar. Thus, it is unlikely that different MAR and MNAR assumptions are the reason for the observed differences in the estimated time effects. We found that WE gave a comparably high time effect estimate. Thus, apparently weighting does not succeed in mimicking the growth levels of the dropout cases appropriately. As compared to FIML, DK, and PM, MI indicated a slightly lower time effect, and WC a slightly higher one. These differences can be explained by the fact that among all of the models considered, only MI and WC acknowledged the multilevel structure of the missing-data model. Both models integrated an individual-specific random effect in the dropout process. WC assumed the same effect for both the analysis and the selection model, whereas MI specified separate ones for the imputation model and the analysis model.

#### Predictors of growth

Students’ reasoning abilities and mathematical self-concept had significant positive effects on mathematical competence (γ_2_ and γ_3_ in Eq. 3) in all considered missing-data models (see the middle panels of Fig. 4). The respective effects were somewhat larger for LWD and WE than for other modeling strategies. Apparently, here issues arise similar to those in the estimation of the intercept. The higher propensity to reparticipate among students with higher self-concept leads to a small overestimation of effects using LWD or WE. Beware of the special nature of the two classes of the PM model. It is obvious that self-concept and reasoning ability affect the competence level more strongly in the class of repeated participants than in the class of students who dropped out. When both classes are considered together, an average effect can be observed similar to those for the other model types (except LWD and WE). In contrast, FIML, MI, and the two selection models converge on similar estimates for the predictors of growth.

The change in mathematical competences over time was larger for students with higher reasoning abilities (γ_4_ in Eq. 3). This finding was rather consistent among all considered models (see the lower right panel of Fig. 4). Only MI and WC, which account for intra-individual variations when dealing with attrition, estimated a smaller effect. In other words, MI and WC facilitate modeling different degrees of heterogeneity among nonresponders and responders. However, as opposed to MI, WC specifies only one effect for both the selection model and the analysis model. Thus, individual heterogeneity concerning the dropout process and concerning competence development might be intermingled. Hence, we deem the estimated time effects of MI to be more reliable than the effects estimated by the other models. In contrast, mathematical self-concept had a negligible impact on changes in mathematical competence (γ_5_ in Eq. 3), with all effects being close to zero. Although some models (FIML, DK, WC) identified minor moderating effects, other models (LWD, MI, WE, PM) found not significant effect at all. Thus, the predominant evidence indicates no effects of students’ self-concept on their change in mathematical competence over time.

#### Random effects

With regard to the random effects (*u* and *v* in Eq. 3), we found surprisingly few differences between the models (see Fig. 5). Variability in mathematical competence between different schools was consistently estimated to be larger than within-subjects variability across time. However, the latter variance was particularly large for WE and comparably small for WC, whereas the reverse was true for the estimated residual variances. That is, WE assigned more variation to the individual level than to the level of competence measurements because the two competence scores of an individual were weighted equally. WC, in contrast, assumes that the same random (latent) factor affects both competence development and the attrition process. Therefore, in the analysis model, less variance is assigned to the individual level and more unexplained variance remains at the residual level.

## Discussion

The presence of selection bias due to nonresponse makes the modeling of competence development a pivotal challenge in longitudinal educational surveys. In our data example, we observed dropout of nearly 60% at the second measurement occasion, which is not uncommon for many voluntary longitudinal surveys such as the NEPS. Statistical analyses need to acknowledge the process governing these missing responses in order to derive unbiased estimates of the longitudinal effects (Enders, 2011; Muthén et al., 2011). We studied the growth of mathematical competence among German adolescents over three years, as well as the effects of reasoning ability and mathematical self-concept thereon. The problem of missing information was addressed by conducting sensitivity analyses with regard to the assumed missing-data model. We estimated different models under MCAR, MAR, and MNAR assumptions to evaluate the robustness of the focal effects of interest. Because each missing-data model makes various (frequently untestable) assumptions concerning the underlying missing-data mechanism or the distributions of unobserved factors, different missing-data models may result in rather different outcomes, a fact that is frequently ignored in the social sciences. For example, assuming a MAR scheme instead of a MNAR mechanism may lead to biased estimates if the dropout pattern (at least partly) depends on the studied outcome (e.g., Example 1 in Little & Wang, 1996; Simulation 1 in Galimard, Chevret, Protopopescu, & Resche-Rigon, 2016). In our data example, we adopted three MAR schemes (i.e., FIML, MI, WE) and three MNAR models (i.e., WC, DK, PM). These models were contrasted with the standard approach for MCAR commonly used in the social sciences, which involves complete case analyses (LD).

Overall, all approaches pointed to similar results concerning the impact of mathematical self-concept and reasoning ability on the change of mathematical competence over three years. Depending on the missing-data model, the effect of mathematical self-concept was very small to nonexistent. Thus, if only a single missing-data model had been estimated (e.g., FIML) one might have concluded that mathematical competence would increase more strongly for students with higher self-concept. However, the comparative results from different missing-data models did not support a robust effect in this regard. Rather, the sensitivity analysis suggested that mathematical self-concept has no pronounced effect on the development of mathematical competence. In contrast, the different models converged on similar conclusions regarding the effects of reasoning: Mathematical competences increased more strongly for students with lower reasoning abilities.

As expected, more pronounced differences between the missing-data models were observed with respect to the mean competence score (i.e., the intercept). Here, listwise deletion and weighting failed, because LWD simply ignored the missing-data problem and WE compensated for the outcome-dependent dropout in a way that distorted the estimated mean competence level. More specifically, in inverse probability weighting, higher weights were assigned to individuals with lower competence scores to compensate for their dropout at the second measurement point. However, this compensation operated at the first measurement point, too, yielding an overestimation of the mean competence score in grade 9.

However, our analyses showed that the consideration of the multilevel structure in a missing-data model can make a difference. Although all estimated time effects indicated a strong increase of competence over time, models that incorporated a multilevel structure in the missing-data model (MI, WC) estimated slightly different effects than did models that disregarded the nested data structure when modeling the dropout process. This effect was also mirrored in the moderating influence of reasoning on the change in mathematical competence. A smaller absolute effect was observed when an individual-specific random effect was specified in the dropout model. The reason for this result might be that for the dropout cases a significant part of the variation in competence growth is explained by individual heterogeneity in the dropout process, and not by pure growth, as would otherwise be assumed. In summary, these analyses demonstrated that the type of missing-data model assumed may lead to research arriving at different conclusions. Applied researchers are, therefore, strongly encouraged to demonstrate the robustness of their results regarding the missing-data model they have adopted.

### Recommendations for longitudinal studies with missing data

Nonresponse is unavoidable in most longitudinal studies that rely on volunteer samples for repeated participation across multiple measurement occasions. In our opinion, several strategies can help improve the validity of inferences drawn from incomplete data:

First and foremost, researchers need to keep in mind the purpose of inference tests: Statistical tests allow for inferences about an unobserved target population from an observed sample. This requires observed samples to be close representations of the target population. However, nonresponse can seriously undermine the validity of population inferences. For example, in case of nonrandom dropout processes and complete case analyses, it is unknown to what degree the observed data still adequately represents the population of interest. Unfortunately, it is still rather uncommon in psychology (and related disciplines) to clearly describe the target population and identify the generalizability of identified effects. Most often results derived from convenient sample—most notably, WEIRD (Western, educated, industrialized, rich, and democratic; Henrich, Heine, & Norenzayan, 2010) samples—are treated as if they could be generalized to anybody around the world. Therefore, we support recent calls for more explicit consideration of the generalizability of research results (Simons, Shoda, & Lindsay, 2017).

Second, the factors driving nonresponse processes need to be scrutinized. Selectivity analyses can help identify variables that explain the participation propensity. This requires taking into account relevant background information on the respondents, such as socioeconomic characteristics, and the survey context, such as the survey mode. Information on the survey experience, such as survey enjoyment or intention to drop out, has been shown to be useful as well (e.g., Leon, Demirtas, & Hedeker, 2007; Rogelberg, Fisher, Maynard, Hakel, & Horvath, 2001).

Third, nonresponse biases are best reduced by including relevant auxiliary variables in the statistical model (Graham, 2009). These can easily be handled with contemporary MAR approaches such as MI or FIML but also in more complex MNAR models (e.g., Diggle & Kenward, 1994; Wu & Carroll, 1988). Good auxiliary variables should predict the missing values, rather than the probability of missingness itself (Little, 1995). Importantly, the model structure for the auxiliary variables should mimic the structure of the analysis model. For example, if a two-level model is the focus of the analyses the two levels of nesting should also be acknowledged in the imputation model. A problem is that higher orders of nesting in missing-data models are frequently not implemented in common software. In this case, it is recommended to focus on the level explaining more variance and ignore less important levels.

Finally, each missing-data model hinges on a number of assumptions—for example, regarding unobserved distributions. Because these assumptions cannot be empirically tested, it is important to evaluate how these assumptions affect the parameter estimates, and consequently the research conclusions. Therefore, we advocate the routine use of sensitivity analyses to estimate different missing-data models, in order to gauge the robustness of the results. We recommend using at least one MAR model, such as MI or FIML, and one MNAR model. These results can be compared to a complete case analysis to evaluate the impact of systematic dropout processes on the results. Our call for systematic sensitivity analyses in longitudinal research with incomplete data echoes similar views put forward for psychological research in general: Any empirical finding should be evaluated with regard to its robustness against flexibilities in data-analytic decisions using, for example, specification-curve analyses (Simonsohn, Simmons, & Nelson, 2015; see Rohrer, Egloff, & Schmukle, 2017, for an application) or multiverse analyses (Steegen, Tuerlinckx, Gelman, & Vanpaemel, 2016). Common to these suggestions and our position on sensitivity analyses in longitudinal research is the notion that researchers should make the assumptions in their statistical models explicit and compare empirical findings across a range of defensible alternative model specifications.

### Open challenges in modeling longitudinal nonresponse

Although methodological research on missing data has substantial increased in recent decades (cf. Carpenter & Kenward, 2012; Enders, 2011; Little et al., 2012; Molenberghs et al., 2008; Muthén et al., 2011), a number of unresolved issues remain that make the acknowledgment of selection bias in longitudinal research challenging. For one, no unique model selection criteria exist that would allow for decisions on which model fits data best. Some of the approaches considered in this article differ structurally to such an extent that standard methods for model comparison (e.g., information criteria or cross-validation) cannot be applied. It is possible to compare, at least approximately, selection models and pattern mixture models using the BIC or AIC. However, FIML and MI differ to such an extent in their likelihood specifications and the numbers of observations used for model estimation that BIC or AIC cannot be applied (see also the discussion in Enders & Mansolf, 2016, on model fit indices in MI). Furthermore, to our knowledge no coherent cross-validation approach currently exists for comparing distinct missing-data models. Thus, at least for the moment, a combination of logical reasoning with comparing estimated effects serves as the only means to assess which of the fitted approaches explains the data properly. Clearly, this strategy becomes inapplicable if the results are very heterogeneous and no logical reason can be found to explain these differences. This is certainly an unsolved problem for which no straightforward solution currently exists.

Another obstacle when dealing with missing-data problems such as the one described in this article is the availability of statistical software to estimate the different missing-data models. We focused on R (R Core Team, 2017), Stata, and Mplus, which are common in the social sciences. All of these packages offer comprehensive functionality for single-level MAR and MNAR problems. However, their functionality with regard to multilevel data structures is somewhat limited. For example, in a single-level setting, the FIML function in Mplus allows for the consideration of auxiliary variables for coping with MAR problems. However, when dealing with three- or higher-level data structures, this functionality is not yet implemented, although Graham’s (2003) saturated-correlates approach should theoretically also be suitable for multilevel structures. Likewise, the mice package in R allows for multiple imputation for two-level data problems, but when it comes to higher levels, no appropriate imputation functions exist (see also Grund et al., 2016). Only recently did Enders, Keller, and Levy (2017) develop a new software program that allows for three-level imputation of the kind needed in educational research. Future research will need to evaluate the implemented imputation algorithm in more detail and compare its accuracy to that of other imputation schemes. Therefore, applied researchers need to be aware of these limitations when adopting the missing-data routines in common statistical software.

### Conclusions

Missing data is a pervasive problem in longitudinal educational research that can bias estimated effects. Because there is no unique and proper way of handling missing data, sensitivity analyses are mandatory in order to reach robust conclusions. This is especially true if an MNAR mechanism is assumed to produce the nonresponse data at hand. Here, sensitivity analyses helped us assess the plausibility of various missing-data mechanisms and evaluate the robustness of their results. For applied researchers, we offer the analysis syntax of our data example in an online repository. We hope that these files can serve as templates for future longitudinal research and help spur the adoption of sensitivity analyses in practice.

#### Author note

This article has used data from the National Educational Panel Study (NEPS): Starting Cohort Grade 9, doi:10.5157/NEPS:SC4:9.0.0. From 2008 to 2013, NEPS data were collected as part of the Framework Program for the Promotion of Empirical Educational Research, funded by the German Federal Ministry of Education and Research (BMBF). As of 2014, NEPS is carried out by the Leibniz Institute for Educational Trajectories (LIfBi) at the University of Bamberg, in cooperation with a nationwide network.

## Electronic supplementary material

ESM 1 (DOCX 55 kb)
