# Supplementary material for: Modeling competence development in the presence of selection bias
Source: Behav Res Methods. 2018 Feb 15;50(6):2426–41. doi: 10.3758/s13428-018-1021-z (PMC6267521; doi:10.3758/s13428-018-1021-z)
Supplement: Supplementary file 1 — (DOCX 55 kb) [file 13428_2018_1021_MOESM1_ESM.docx]

# S1. Formal description of the MNAR models DK, WC, and PM

The joint distribution of the focal model in [3] and the missing data indicator for individual $i$in school *s* at the time points *t=1,2*is

[*MATH_i_*, *R_i_*, θ*_i_* | *X_i_*], [S1]

where *MATH_i_* comprises *MATH_1is_*and *MATH_2is_*, *R_i_* is 1 if we have no observation for the second measurement point and 0 otherwise, and θ*_i_*comprises the fixed effects γ_1_ = [γ_1_, …, γ_5_]. The two random effects *u_i_* and *v_s_* have variances of *Var*(*u_i_*) = σ*_u_*^2^ and *Var*(*v_s_*) = σ*_v_*^2^, respectively. *X_i_* consists of the observed data points for each student, thus, *X_i_* = [1, *TIME_tis_*, *REAS_is_*, *SC_is_*, *TIME_tis_*∙*REAS_tis_*, *TIME_tis_*∙*SC_tis_*]*_t_*_=1,2_.

The distribution in [S1] can be factorized intoa selection model representation as

[*MATH_i_*, *R_i_*, θ*_i_* | *X_i_*] = [*MATH_i_* |*X_i_*, θ*_i_*] ∙ [*u_i_*, *v_s_* | *X_i_*]∙ [*R_i_* | *X_i_, MATH_i_*,θ*_i_*]. [S2]

In our case, [*MATH_i_* | *X_i_*, θ*_i_*] = *N*_2_(α*_i_*, Σ) with α*_i_* = *X_i_∙*γ + *u_i_* + *v_s_* and $\Sigma=\left(\begin{matrix} \sigma_{\varepsilon}^{2}+\sigma_{u}^{2}+\sigma_{v}^{2} & \sigma_{u}^{2}+\sigma_{v}^{2} \\ \sigma_{u}^{2}+\sigma_{v}^{2} & \sigma_{\varepsilon}^{2}+\sigma_{u}^{2}+\sigma_{v}^{2} \end{matrix}\right)$. If [*R_i_* | *X_i_, MATH_i_*,θ*_i_*] = [*R_i_* | *X*] the missingness pattern is MAR and FIML, MI, or weighting may be used for statistical inference. Otherwise, when facing outcome dependent dropout (as suspected in our case), [*R_i_* | *X_i_, MATH_i_*,θ*_i_*] has to be modeled, for example, as the outcome of a Bernoulli experiment, [*R_i_* | *X_i_, MATH_i_*,θ*_i_*] = *B*(*p_i_*) with *p_i_* = *Pr*(*R_i_* = 1 | *MATH_i_*, *X_i_*). For this purpose, Diggle and Kenward (1994) suggested using a logit model of the form *p_i_* = logit(β_0_ + β_0_∙*MATH*_1_*_is_* + β_0_∙*MATH*_2_*_is_*). In an extended model version, they made β_0_ a function of covariates and external factors. For our application, we used this model version and assumed that β_0_ is a function of *X_i_* and the selection variables found to affect the response behavior. Note that setting β_2_= 0 yields a MAR model. Wu and Carroll (1988) added a frailty term accounting for individual heterogeneity in the dropout process, resulting in *p_i_* = probit(β_0_ + β_0_∙*MATH*_1_*_is_* + β_0_∙*MATH*_2_*_is_* + *u_i_*). They assumed the same frailty term for the focal and the missing data model. In contrast to Diggle and Kenward (1994), Wu and Carroll (1988) used a probit regression to map the dropout process.

Pattern mixture models factorize the distribution in [S1] differently (Little, 1995) as

[*MATH_i_*, *R_i_*, θ*_i_* | *X_i_*] = [*MATH_i_* |*X_i_*, *R_i_*, θ*_i_*] ∙ [*u_i_*, *v_s_* | *X_i_, R_i_*]∙ [*R_i_* | *X_i_*], [S3]

where [*MATH_i_* |*X_i_*, *R_i_*, θ*_i_*] models the individual regression line separately for the two dropout patterns *R_i_* = 0 and *R_i_* = 1. [*u_i_*, *v_s_* | *X_i_, R_i_*] is the distribution of the individual- and school specific effects depending on*X_i_*and *R_i_*. [*R_i_* | *X_i_*],[*R_i_* | *X_i_*] gives the marginal proportion of each of the two missingness patterns as a function of*X_i_*. The two dropout patterns *R_i_* = 0 and *R_i_* = 1 result in a mixture of two normal distributions for *MATH_i_*, [*MATH_i_* |*X_i_*, *R_i_* = 0, θ*_i_*] = *N*_2_(α*_i_*^(1)^, Σ) and [*MATH_i_* |*X_i_*, *R_i_* = 1, θ*_i_*] = *N*_2_(α*_i_*^(2)^, Σ), with α*_i_*^(1)^ =(*X_i_*∙ γ^(1)^ + *u_i_* + *v_s_*)*_t_* = 1, 2, α*_i_*^(2)^ =(α_1_*_i_*^(2)^, α_2_*_i_*^(2)^), α_1_*_i_*^(2)^ =(*X_i_*∙ γ^(2)^ + *u_i_* + *v_s_*)*_t_* _= 1_, and α_2_*_i_*^(2)^ = γ_0_^(2)^+γ_2_^(2)^∙ *REAS_is_* + γ_3_^(2)^∙ *SC_is_*+ *u_i_* + *v_s_*. The missing data mechanism is MCAR if [*MATH_i_* |*X_i_*, *R_i_* = 0, θ*_i_*] = [*MATH_i_* |*X_i_*, *R_i_* = 1, θ*_i_*]. In case of MNAR, the estimation of the pattern mixture model requires restrictions on the nature of the missing data mechanism or prior information on [*MATH_2is_* |*MATH_1is_,R_i_* = 1]. Otherwise, the model is not identifiable. In the considered case, we fixed predictors of growth to be zero for *R_i_* = 1and estimated the marginal proportion [*R_i_* | *X_i_*] using a logit regression.

Table S2.
*Estimated Regression Coefficients with 95% Confidence Intervals for MCAR Model.*

|  | | Listwise deletion (LWD) | | | | | |
| --- | --- | --- | --- | --- | --- | --- | --- |
|  | | *B* | 95% CI | | | | |
| Intercept | | 0.443 | | [0.300,0.585] | | | | |
| Time^a^ | | 0.532 | | [0.484,0.581] | | | | |
| Reasoning | | 0.309 | | [0.257,0.361] | | |
| Self-concept | 0.410 | | | | [0.362,0.459] | | | | |
| Time x reasoning | -0.137 | | | | [-0.187,-0.087] | | | | |
| Time x self-concept | -0.015 | | | | [-0.065,0.035] | | | | |
| Random Effect: individual | 0.329 | | | | | [0.308,0.348] | | | | |
| Random Effect: school | 0.328 | | | | | [0.312,0.357] | | | | |
| Residual variance | 0.421 | | | | | [0.407,0.435] | | | | |
| *Note*. ^a^ Coded as 0 = Grade 9 (in 2010)and 1 = students in 2013. Reasoning and self-concept were *z*-standardized. | | | | | | | |

Table S3.
*Estimated Regression Coefficients with 95% Confidence Intervals forMAR Models.*

|  | Full information maximum likelihood (FIML) | | Inverse probability weighting (WE) | | Multivariate imputation via chained equations (MI) | |
| --- | --- | --- | --- | --- | --- | --- |
|  | *B* | 95% CI | *B* | 95% CI | *B* | 95% CI |
| Intercept | -0.067 | [-0.122,-0.012] | 0.189 | [0.110,0.268] | -0.066 | [-0.119,-0.013] |
| Time ^a^ | 0.610 | [0.586,0.635] | 0.798 | [0.745,0.851] | 0.534 | [0.507,0.562] |
| Reasoning | 0.274 | [0.257,0.290] | 0.329 | [0.290,0.368] | 0.279 | [0.261,0.296] |
| Self-concept | 0.347 | [0.332,0.362] | 0.382 | [0.349,0.417] | 0.345 | [0.330,0.360] |
| Time x reasoning | -0.128 | [-0.154,-0.102] | -0.111 | [-0.162,-0.059] | -0.048 | [-0.069,-0.026] |
| Time x self-concept | 0.022 | [0.000,0.045] | 0.025 | [-0.012,0.063] | 0.011 | [-0.008,0.030] |
| Random Effect: individual | 0.277 | [0.261,0.294] | 0.478 | [0.454,0.504] | 0.296 | [0.288,0.294] |
| Random Effect: school | 0.389 | [0.338,0.440] | 0.330 | [0.287,0.379] | 0.357 | [0.362,0.368] |
| Residual variance | 0.399 | [0.385,0.413] | 0.191 | [0.173,0.210] | 0.409 | [0.414,0.427] |
| *Note*. ^a^Coded as 0 = Grade 9 (in 2010) and 1 = students in 2013. Reasoning and self-concept were *z*-standardized. | | | | | | | |

Table S4.
*Estimated Regression Coefficients with 95% Confidence Intervals for MNAR Models.*

|  | Diggle-Kenward selection model (DK) | | Wu-Carroll selection model (WC) | | Pattern mixture model All-time participants (PM1) | | Pattern mixture model Dropouts (PM0) | |
| --- | --- | --- | --- | --- | --- | --- | --- | --- |
|  | *B* | 95% CI | *B* | 95% CI | *B* | 95% CI | *B* | 95% CI |
| Intercept | -0.075 | [-0.129,-0.021] | -0.069 | [-0.141,0.002] | 0.078 | [0.019,0.136] | -0.166 | [-0.219,-0.112] |
| Time ^a^ | 0.545 | [0.567,0.590] | 0.549 | [0.526,0.572] | 0.578 | [0.553,0.603] | 0.000^†^ | [0.000,0.000] |
| Reasoning | 0.279 | [0.263,0.296] | 0.277 | [0.263,0.290] | 0.337 | [0.308,0.365] | 0.241 | [0.222,0.261] |
| Self-concept | 0.346 | [0.332,0.361] | 0.339 | [0.326,0.352] | 0.410 | [0.387,0.433] | 0.289 | [0.270,0.308] |
| Time x reasoning | -0.117 | [-0.141,-0.093] | -0.076 | [-0.100,-0.051] | -0.158 | [-0.186,-0.129] | 0.000^†^ | [0.000,0.000] |
| Time x self-concept | 0.037 | [0.016,0.058] | 0.048 | [0.025,0.070] | -0.015 | [-0.04,0.010] | 0.000^†^ | [0.000,0.000] |
| Random Effect: individual | 0.257 | [0.242,0.273] | 0.105 | [0.097,0.113] | 0.272^‡^ | [0.255,0.288] | 0.272^‡^ | [0.255,0.288] |
| Random Effect: school | 0.377 | [0.328,0.426] | 0.370 | [0.304,0.436] | 0.332^‡^ | [0.287,0.376] | 0.332^‡^ | [0.287,0.376] |
| Residual variance | 0.425 | [0.411,0.439] | 0.584 | [0.573,0.595] | 0.397^‡^ | [0.383,0.411] | 0.397^‡^ | [0.383,0.411] |
| *Note*. ^a^ Coded as 0 = Grade 9 (in 2010) and 1 = students in 2013. Reasoning and self-concept were *z*-standardized. ^†^ Fixed parameter by design.  ^‡^ Because of identification purposes, variances are assumed to be equal for PM1 and PM0 | | | | | | | | |
